# Development of CAD based on ANN analysis of power spectra for pneumoconiosis in chest radiographs: effect of three new enhancement methods

**DOI:** 10.1007/s12194-013-0255-9

**Published:** 2014-01-12

**Authors:** Eiichiro Okumura, Ikuo Kawashita, Takayuki Ishida

**Affiliations:** 1 Department of Medical Radiological Technology, Kagoshima Medical Technology College, 5417-1, Hirakawa, Kagoshima 891-0133 Japan; 2 Department of Clinical Radiology, Hiroshima International University, 555-36, Kurosegakuendai, Higashihiroshima, Hiroshima 739-2695 Japan; 3 Division of Health Sciences, Graduate School of Medicine, Osaka University, 1-7, Yamadaoka, Suita, 565-0871 Japan

**Keywords:** Computer-aided diagnosis (CAD), Pneumoconiosis, Chest radiography, Power spectra, Artificial neural network

## Abstract

We have been developing a computer-aided detection (CAD) scheme for pneumoconiosis based on a rule-based plus artificial neural network (ANN) analysis of power spectra. In this study, we have developed three enhancement methods for the abnormal patterns to reduce false-positive and false-negative values. The image database consisted of 2 normal and 15 abnormal chest radiographs. The International Labour Organization standard chest radiographs with pneumoconiosis were categorized as subcategory, size, and shape of pneumoconiosis. Regions of interest (ROIs) with a matrix size of 32 × 32 were selected from normal and abnormal lungs. Three new enhanced methods were obtained by window function, top-hat transformation, and gray-level co-occurrence matrix analysis. We calculated the power spectrum (PS) of all ROIs by Fourier transform. For the classification between normal and abnormal ROIs, we applied a combined analysis using the ruled-based plus the ANN method. To evaluate the overall performance of this CAD scheme, we employed ROC analysis for distinguishing between normal and abnormal ROIs. On the chest radiographs of the highest categories (severe pneumoconiosis) and the lowest categories (early pneumoconiosis), this CAD scheme achieved area under the curve (AUC) values of 0.93 ± 0.02 and 0.72 ± 0.03. The combined rule-based plus ANN method with the three new enhanced methods obtained the highest classification performance for distinguishing between abnormal and normal ROIs. Our CAD system based on the three new enhanced methods would be useful in assisting radiologists in the classification of pneumoconiosis.

## Introduction

Pneumoconiosis has relatively specific radiographic features, such as diffuse lung parenchyma lesions. Pneumoconiosis includes asbestosis, silicosis, and other occupational diseases caused by exposure to dust [1]. Pneumoconiosis may be classified as either fibrotic or nonfibrotic, according to the presence or absence of fibrosis [2]. Siderosis, stannosis, and baritosis are the nonfibrotic forms of pneumoconiosis that result from inhalation of iron oxide, tin oxide, and barium sulfate particles, respectively [2]. The International Labour Organization (ILO) has established a standardized system for classifying radiographic abnormalities in pneumoconiosis based on the presence of the following lung parenchymal and pleural abnormalities: small rounded opacities, small irregular opacities, and profusion of opacities [2–4].

The radiographic changes in some cases of the initial reticular forms of pneumoconiosis are difficult to diagnose [2, 5]. Pleural plaque on plain chest radiographs, mimicking shadows, such as rib-companion shadows, may lead to misclassification of conditions consistent with pneumoconiosis [1]. Therefore, computer-aided diagnosis (CAD) systems for chest radiographs are potentially useful tools that can lead to a more accurate diagnosis of various lung diseases [6–17]. For the computerized detection of interstitial lung disease on chest radiographs, a number of researchers have developed CAD schemes based on the Fourier transform [6–8], geometric-pattern feature analysis [9], and artificial neural network (ANN) analysis [10] of image data. In addition, CAD systems for diffuse lung disease on thoracic computed tomography (CT) have been developed. These CAD schemes were based on histogram features [12], the run length matrix (RLM) [12], the gray-level co-occurrence matrix (GLCOM) [12], the Gaussian filter bank-based method [13], morphologic filter-based feature analysis [14], subjective clinical features provided by radiologists [15], a hybrid of three single networks with expert rules [16], and adaptive multiple-feature methods [17]. On the other hand, CAD systems for detection of pneumoconiosis on chest radiographs have been developed for improved detection performance by radiologists [18–28]. Use of a combination of a multi-scale difference filter bank with histogram and GLCOM for extracting discriminatory features from each zone, the utility of a support vector machine (SVM) as a region-level classifier and the employment of a chest-level classifier to incorporate six regions’ prediction results in the final classification [22].

Thus, many researchers [6–27] obtained a specific index with textural features and used discrimination analysis such as ANN and SVM for distinction between normal and abnormal lungs. Therefore, because we obtained more information on abnormal and normal lungs, we developed a CAD system for the distinction between normal and abnormal patterns in pneumoconiosis using the ANN trained with the power spectrum (PS) values [28].

However, according to the subcategory, size, and shape with standard radiographs and the guideline defined by the ILO and Ministry of Labor, radiologists subjectively classify category. The profusion level of small opacities can reflect the degree of pneumoconiosis. It is difficult for radiologists to classify pneumoconiosis with small and irregular opacities on chest radiographs. In addition, for recognizing handicap, it was necessary for radiologists to correctly classify category.

Therefore, we have been engaged in the development of a CAD scheme for pneumoconiosis with each subcategory, size, and shape using rule-based plus ANN analysis of the PS with three new enhancement methods for the abnormal patterns to reduce false positives and false negatives. In addition, we investigated the effects of various parameters on the overall classification performance.

## Materials and methods

### Materials

**Fig. 1:**
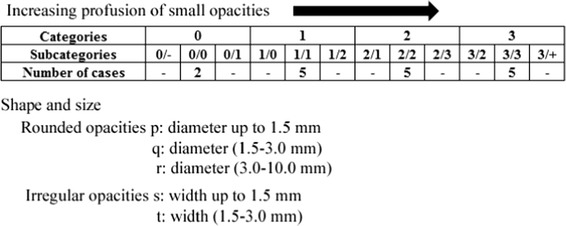
ILO classification scheme for small opacities in pneumoconiosis

Figure 1 shows the ILO classification scheme for small opacities in pneumoconiosis. The small opacities was divided into four categories, ranging from a completely normal lung (category 0) to severe pneumoconiosis (category 3). Our image database consisted of two normal and 15 abnormal posteroanterior (PA) chest radiographs. The two normal cases define subcategory 0/0, and 15 abnormal cases, respectively, define subcategories 1/1, 2/2, and 3/3 with some of the shapes and sizes of these opacities (*p*, *q*, *r*, *s*, and *t* in Fig. 1; Table 1). These images were digitized with a pixel size of 0.175 mm, a matrix size of 2468 × 2034, and 12-bit depth. The profusion of small opacities refers to the concentration of small opacities in the affected zones of the lung [4]. Classification of a radiograph using the 12-subcategory scale (between subcategories 0/− and 3/+ in Fig. 1) was performed [4]. The appropriate category was chosen by comparison of a subject radiograph with standard radiographs that define the levels of profusion characteristic of the subcategories (0/0, 1/1, 2/2, 3/3) within these categories (0, 1, 2, and 3) [4]. The category was recorded by writing the corresponding symbol followed by an oblique stroke, i.e. 0/, 1/, 2/, 3/ [4]. If no alternative category was seriously considered, the radiograph was classified in the subcategory, i.e. 0/0, 1/1, 2/2, 3/3 [4].

**Table 1:** ILO classification scheme for rounded and irregular opacities on subcategories and the number of ROIs on (a) subcategory 0/0 and (b) subcategories 1/1, 2/2, and 3/3

	No. of cases	
a		
Subcategory 0/0	Case 1	Case 2
Number of ROIs	101	96

	Shape and size				
	Rounded opacities			Irregular opacities	
b					
Subcategory 1/1	*p*/*p*	*q*/*q*	*r*/*r*	*s*/*t*	*t*/*t*
Number of ROIs	154	124	100	117	101
Subcategory 2/2	*p*/*p*	*q*/*q*	*r*/*r*	*s*/*s*	*t*/*t*
Number of ROIs	144	92	152	159	105
Subcategory 3/3	*p*/*p*	*q*/*q*	*r*/*r*	*s*/*s*	*t*/*t*
Number of ROIs	226	122	176	101	169

The opacities were also classified by size and shape, as either rounded or irregular opacities. In each case, three sizes were differentiated. For small rounded opacities, the three size ranges were denoted by the letters p, q, and r, and were defined by the appearances of the small opacities on the corresponding standard radiographs (Fig. 1) [4]. When small opacities of different shapes and/or sizes were seen, the letter for the predominant shape and size (primary) was recorded before the oblique stroke, whereas the letter for the less frequently occurring shape and size (secondary) was recorded after the oblique stroke [4].

### Overall classification schemes with combined rule-based plus ANN method

**Fig. 2:**
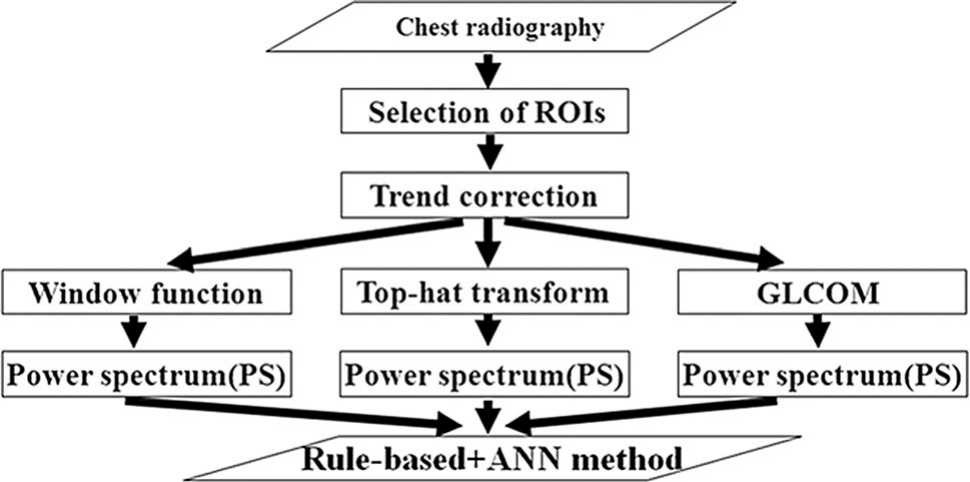
The overall classification scheme with combined rule-based plus ANN method

Figure 2 shows the overall classification scheme with the combined rule-based plus ANN method with the use of three new enhancement methods. First, the regions of interest (ROIs) with a matrix size of 32 × 32 pixels were manually selected from normal and abnormal cases in intercostal spaces and over rib spaces by an experienced radiological technologist [7]. We eliminated overlap with ROIs. Table 1 shows the number of ROIs on each case. We obtained a trend correction in selected ROIs using a two-dimensional surface-fitting technique based on the least-square method because pixel values were different between the gross anatomy of the lung and chest wall regions on chest radiographs [7].

We performed a trend correction with second-order polynomial surfaces. Three new enhancement methods, a window function image, top-hat transform image, and GLCOM feature image, were applied to trend-correction images. The effects of the window function image, top-hat transform image, and GLCOM feature image will be discussed later. In these three enhancement methods, we calculated a PS of all ROIs by Fourier transform [28]. We used only PS values on the main and second axes, which have the maximum and the second maximum PS values on the radial line from the center of the PS image, respectively [28]. We used the PS values on the positive main and second axes that represented spatial frequency because they were symmetric to the center of the PS image [28]. For classification between normal and abnormal ROIs, we applied a combination of the ruled-based plus ANN method of the PS value with these three enhancement methods.

### Reduction of high-frequency distortion with window function

**Fig. 3:**
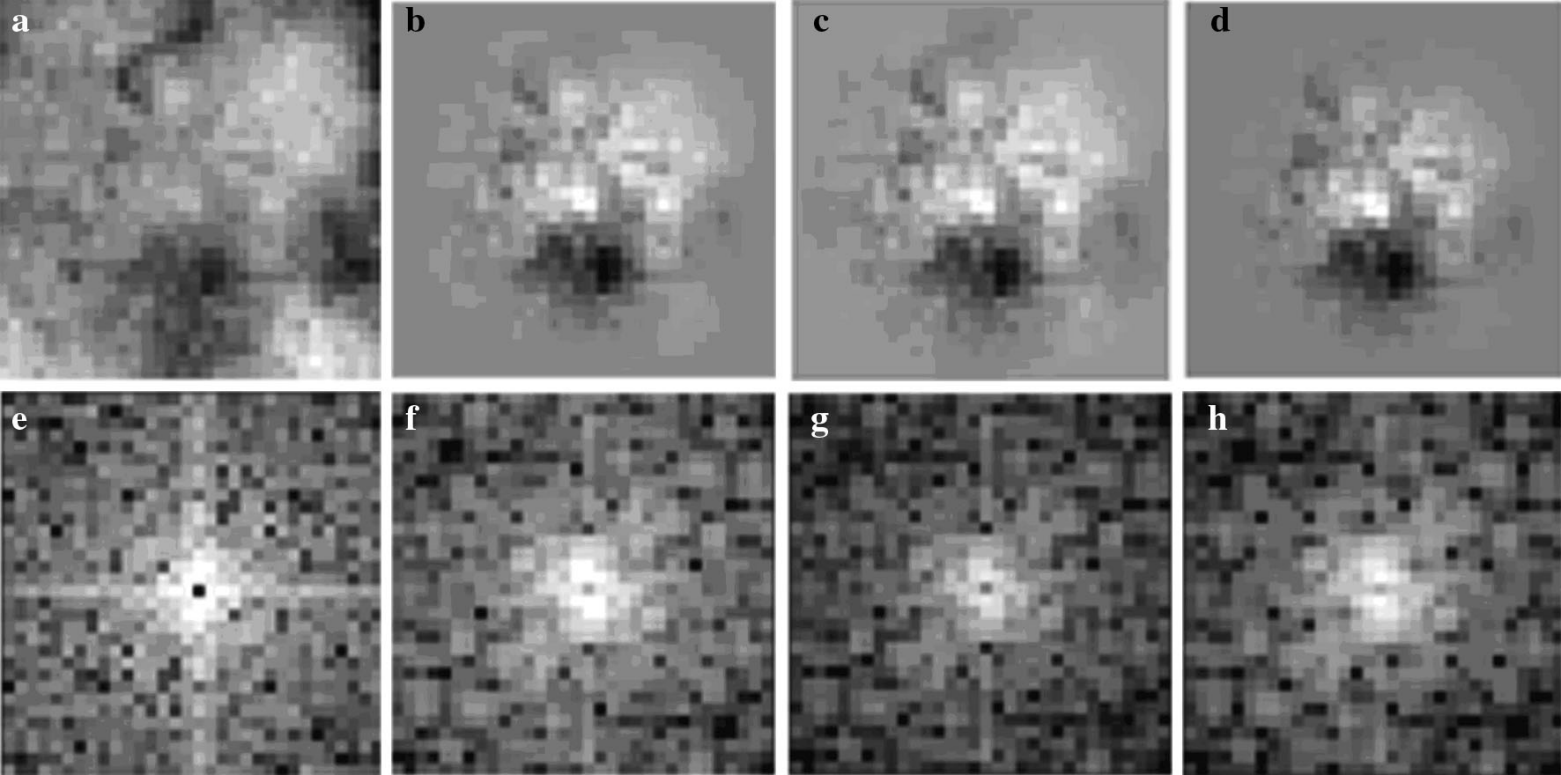
Window function images. **a** Trend-correction image, **b** the Hamming window function image, **c** the Hanning window function image, and **d** the Blackman window function image. **e**–**h** The PS images of images **a**–**d**

With regard to the effect of a noncontiguous pixel value on the edge of the ROI, higher PS values appeared on the *u* and *v* axes that represented spatial frequency. Therefore, it is necessary for the edge of the ROI to be smoother than the central point of the ROI and to extract the original frequency of the ROI. Therefore, we obtained a Hanning, Hamming, and Blackman window function as the window function. These window function images were applied to the trend-correction images. The Hanning (*H*(*n*)), Hamming (*h*(*n*)), and Blackman (*B*(*n*)) window function images were defined as follows:

$$H(n) = 0.5 + 0.5\cos \frac{2\pi n}{N - 1},$$

$$h(n) = 0.54 + 0.46\cos \frac{2\pi n}{N - 1},$$

$$B(n) = 0.42 + 0.5\cos \frac{2\pi n}{N - 1} + 0.08\cos \frac{4\pi n}{N - 1},$$

where *N* is the number of data and *n* ( $- \frac{N - 1}{2} \le n \le \frac{N - 1}{2}$) is the position of the data. We calculated $n = \sqrt {x^{2} + y^{2} }$on two-dimensional spatial frequency domains. We calculated the PS of all ROIs by Fourier transform. The spatial frequency was increased in the order of the Hamming, Hanning, and Blackman window function image. In contrast, the dynamic range was decreased in the order of the Blackman, Hanning, and Hamming window function images. As shown in Fig. 3, on the PS image of the trend-correction image, high PS values were seen on the *u* and *v* axes. However, on the PS image of each window function image, the high PS values resulting from the edge of the ROI were removed. The effect of each window function image on the classification performance will be discussed later.

### Enhancement method with the top-hat transform

**Fig. 4:**
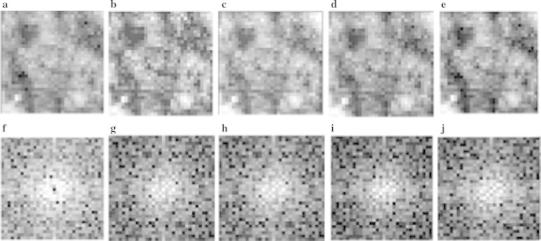
Top-hat transform images. **a** Trend-correction image, **b** top-hat transform image (13 × 13 pixels), **c** top-hat transform image (17 × 17 pixels), **d** top-hat transform image (21 × 21 pixels), and **e** top-hat transform image (25 × 25 pixels). **f**–**j** The PS images of images **a**–**e**

As the opening processing of trend-correction images with a flat structuring element removed peaks and ridges from the topographic surface, a rough background element alone on the trend-correction image remained. A morphologic top-hat transform produced the hollows and ravines of the topographic surface of the trend-correction image. The morphologic top-hat transform is defined by subtraction of the opening processing of a trend-correction image from the trend-correction image [14]. The top-hat transform images with structure elements from 13 × 13 to 25 × 25 pixels were applied to the trend-correction images, so that nodular and irregular opacities could be extracted and large vessels were removed (Fig. 4a–e). We calculated the PS of all ROIs by Fourier transform (Fig. 4f–j). The effect of structure element pixels on the classification performance with the top-hat transform will be discussed later.

### Enhancement method with gray-level co-occurrence matrix (GLCOM) feature image

**Fig. 5:**
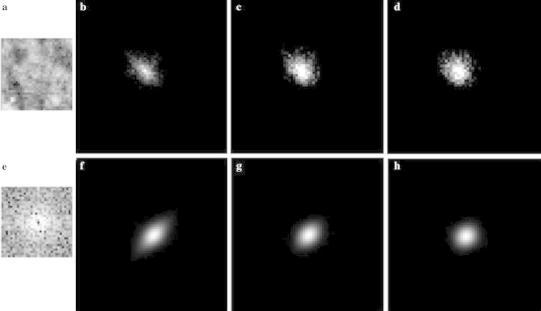
GLCOM feature images. **a** Trend correction, **b** GLCOM feature image (distance of 1 pixel), **c** GLCOM feature image (distance of 2 pixels), and **d** GLCOM feature image (distance of 3 pixels). **e**–**h** The PS images of images **a**–**d**

The GLCOM feature image is a well-established tool for characterizing the spatial distribution of gray levels in an image [26]. An element of the GLCOM feature image was defined by the number of pairs of pixel values separated by a given distance in a direction. If two pixel values are different, an element of a GLCOM measures the “changes” in gray levels [26]. An element at location (*i*, *j*) of the co-occurrence matrix signifies the joint probability density of the occurrence of gray levels *i* and *j* in a specified direction *θ* (*θ* = 45°, 225°) and at a specified distance *d* from each other [26]. GLCOM feature images were applied to the trend-correction images on a 6-bit depth (Fig. 5a–d). The horizontal and vertical directions of the GLCOM feature images denoted the number of gray levels. We calculated the PS of all ROIs by Fourier transform (Fig. 5e–h). The effect of the distance level on the classification performance with the GLCOM feature image alone will be discussed later. In addition, the classification performance with the GLCOM feature image was affected by bit depth. Therefore, to investigate the effects of the bit depth with the rule-based plus ANN method, we varied the gray-level scales from 12- to 4-bit depth.

### Effects of various parameters

In this study, we investigated the effects of many parameters used in the rule-based plus ANN method [28]. We randomly divided the data into abnormal ROIs and normal ROIs for each case, and we obtained training data and non-training data. First, we examined the effects of the Hamming, Hanning, and Blackman window function. A rule-based method using PS values at 0.179 and 0.357 cycles per millimeter, corresponding to the spatial frequencies of nodular patterns, was used for identification of obviously normal or obviously abnormal ROIs [28]. Then, the ANN method was applied for classification of the remaining ROIs, which were not classified as obvious ROIs by the rule-based method. In the rule-based method, if the values for the abnormal ROIs were higher than that of the maximum normal ROI, these abnormal ROIs were classified as “obviously” abnormal. If the values for the normal ROIs were lower than that of the minimum abnormal ROI, they were classified as “obviously” normal.

The ANN method was composed of three units consisting of input, hidden, and output layers. The ANN method with the window function and top-hat transform was composed of 32 input units, 17 hidden units, and one output unit. The ANN method with the GLCOM feature image was composed of 64 input units, 33 hidden units, and one output unit. It is important to note that training with “0.1” for normal patterns and “0.9” for abnormal patterns was intended to distinguish between the abnormal and normal ROIs using the ANN method. Finally, the average classification performance for the ANN method alone was determined using ten different training data and non-training data sets.

To investigate the effects of structure elements on the top-hat transform, we varied the structure element from 13 × 13 pixels to 25 × 25 pixels. As discussed above, we studied the classification performance between normal and abnormal ROIs using the rule-based plus ANN method with the top-hat transform alone. In addition, to investigate the effects of the distance level on the GLCOM feature image, we varied the distance level from 1 to 3 pixels. As discussed above, we studied the classification performance between normal and abnormal ROIs using the rule-based plus ANN method with the GLCOM feature image alone.

### Evaluation of classification performance on overall classification schemes with combined rule-based plus ANN method

**Fig. 6:**
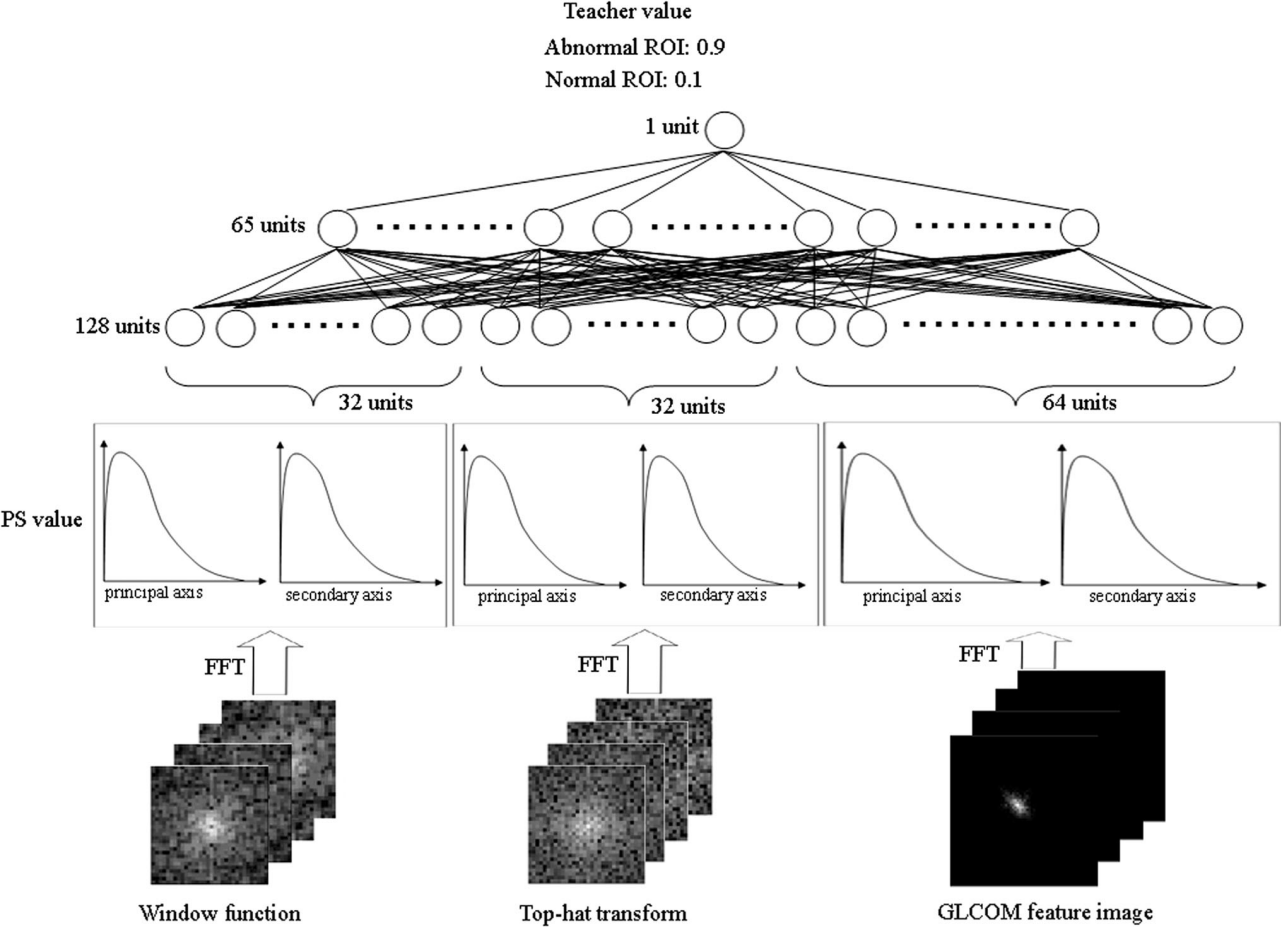
Combined ANN method with window function, top-hat transform, and GLOCM feature image

After we decided various parameters on window function, top-hat transform, and GLCOM feature image for each case used in the rule-based plus ANN method, to improve the classification performance with each of the three enhancement methods, we applied a combined scheme based on the window function, top-hat transform, and GLCOM feature image for distinction between normal and abnormal ROIs using a combined rule-based plus ANN method. In the combined rule-based method in which the combined analysis of the window function, top-hat transform, and GLCOM feature image was used, abnormal ROIs were classified by the logical OR operation, (if the ROI could be classified as abnormal by window function, top-hat transform, or GLCOM feature image, the ROI was finally classified as an obviously abnormal ROI. Obviously normal ROI was classified in a similar way). In the combined ANN method, as shown in Fig. 6, the ANN was composed of 128 input units, 65 hidden units, and one output unit. The input data of the window function and top-hat transform each consisted of a total of 32 pieces (16 normalized PS values for each of the main and second axes). The GLCOM input data consisted of 64 pieces (32 normalized PS values for each of the main and second axes). Thus, input data with the combined ANN method consisted of 128 pieces. It is important to note that training with “0.1” for normal patterns and “0.9” for abnormal patterns was intended to distinguish between the abnormal and normal ROIs using the combined ANN method.

To evaluate the overall performance of the combined rule-based plus ANN method, we employed receiver operating characteristic (ROC) analysis to distinguish between normal and abnormal ROIs. As is the case in the rule-based plus ANN method, we randomly divided the data into abnormal ROIs (Table 2) and normal ROIs (197 ROIs) for each case, and we created ten different pairs of data sets for training and testing. The ROC curve was obtained by averaging of the ROC curves derived from the ten different pairs of data sets. The statistical significance of differences between ROC curves was determined by applying the two-tailed paired *t* test to the AUC (area under the ROC curve) of each test data set.

**Table 2:** The number of ROIs on training data and non-training data

	Shape and size				
	Rounded opacities			Irregular opacities	
Subcategory 1/1	*p*/*p*	*q*/*q*	*r*/*r*	*s*/*t*	*t*/*t*
Training data	77	62	50	59	51
Non-training data	77	62	50	58	50
Subcategory 2/2	*p*/*p*	*q*/*q*	*r*/*r*	*s*/*s*	*t*/*t*
Training data	72	46	76	80	53
Non-training data	72	46	76	79	52
Subcategory 2/2	*p*/*p*	*q*/*q*	*r*/*r*	*s*/*s*	*t*/*t*
Training data	113	61	88	51	85
Non-training data	113	61	88	50	84

## Results and discussion

### Effects of the window function alone with the rule-based plus ANN method

**Fig. 7:**
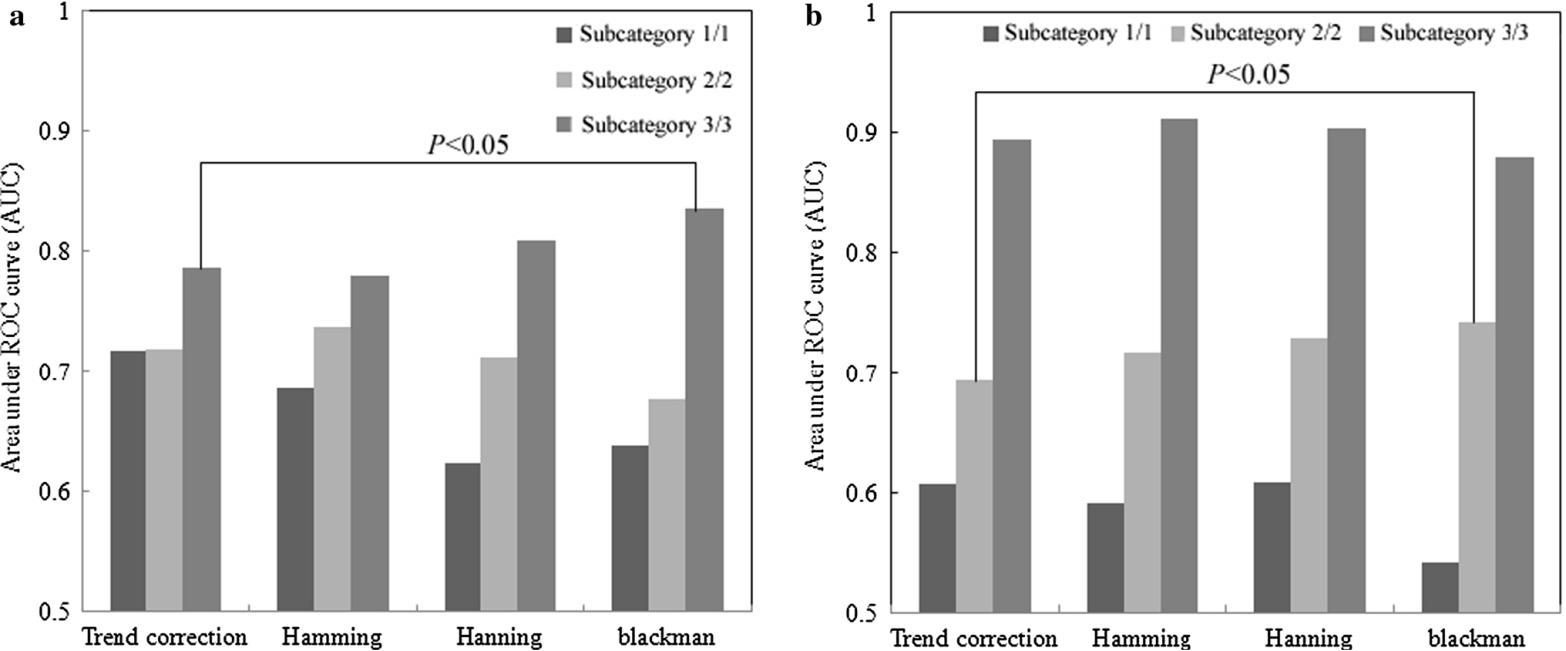
Effect of the window function on each subcategory of small irregular opacities with sizes of *s*/*s* (**a**) and *t*/*t* (**b**)

The effects of the window function on each subcategory of small irregular opacity with a size of *s*/*s* are shown in Fig. 7a. The AUC value for subcategory 3/3 was 0.84 ± 0.03 with the Blackman window function image, which was larger than the value of 0.79 ± 0.04 with the trend-correction image (*P* < 0.05). The effects of the window function on each subcategory of small irregular opacity with a size of *t*/*t* are shown in Fig. 7b. The AUC value for subcategory 2/2 was 0.74 ± 0.05 with the Blackman window function image, which was larger than the value of 0.69 ± 0.05 with the trend-correction image (*P* < 0.05). There was no statistical significance with other cases. As in the previous study of Katsuragawa et al. [27] regarding the relationship between the first moment of the PS and the root-mean-square (RMS) variation, the first moment of the PS of subcategory 2/2 with a size of *t*/*t* was similar to that of subcategory 3/3 with a size of *s*/*s*. As the frequency on this first moment of PS was similar to that of the Blackman window function, the classification performance between normal and abnormal ROIs with the Blackman window function was increased to a greater extent compared to that with the trend-correction image alone. Therefore, the Blackman window function showed a better classification performance on subcategory 2/2 with a size of *t*/*t* and subcategory 3/3 with a size of *s*/*s* compared to the trend-correction image alone.

### Effects of the top-hat transform alone with the rule-based plus ANN method

**Fig. 8:**
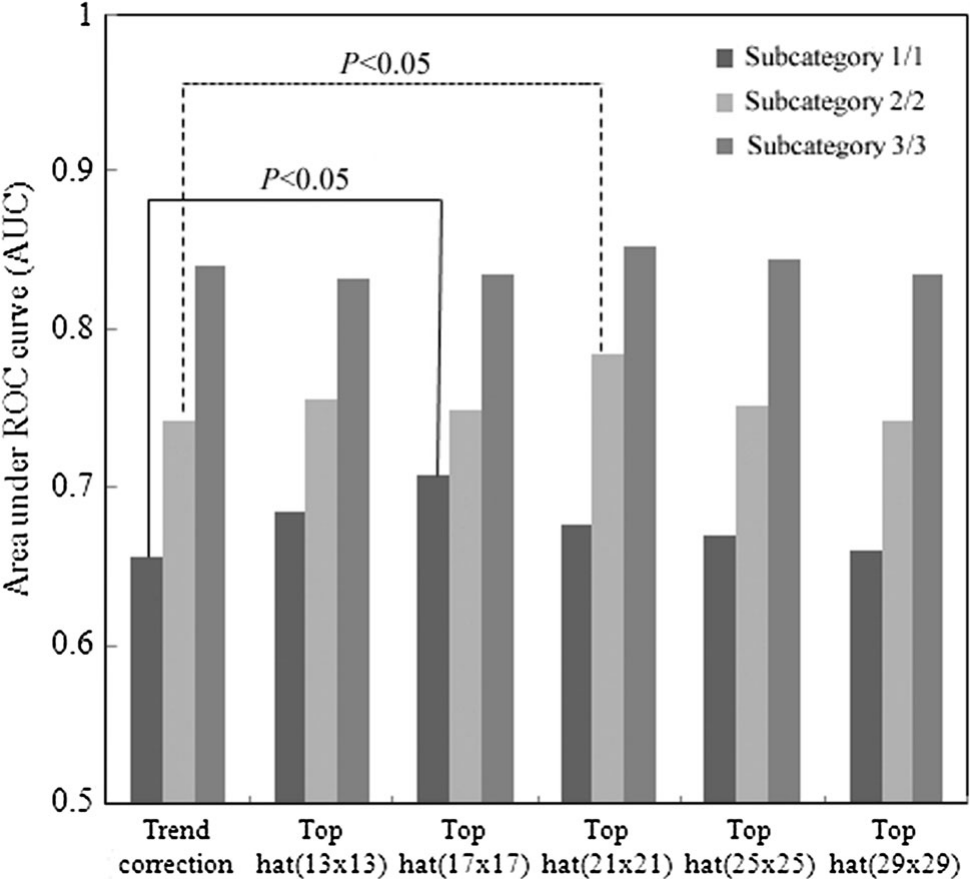
Effects of structure element of the top-hat transform on all small rounded and irregular opacities

The effects of structure elements on the top-hat transform are shown in Fig. 8. The AUC value for subcategory 2/2 was 0.78 ± 0.05 with the top-hat transform (21 × 21 pixels), which was larger than the value of 0.74 ± 0.06 with the trend-correction image (*P* < 0.05). The AUC value for subcategory 1/1 was 0.71 ± 0.05 with the top-hat transform (17 × 17 pixels), which was larger than the value of 0.66 ± 0.06 with the trend-correction image (*P* < 0.05). There was no statistical significance with three cases in subcategory 3/3. The distance between small opacities on subcategory 3/3 was shorter than that for subcategory 1/1. In addition, the profusion of small opacities in subcategory 3/3 was more concentrated than that in subcategory 1/1. A small structure element of opening processing corresponded to a large distance between small opacities as subcategory 1/1. This result suggested that the structure element of 17 × 17 pixels in subcategory 1/1, that of 21 × 21 pixels in subcategory 2/2, and no top-hat transform (trend-correction image) in subcategory 3/3 may correspond to the distance between small opacities.

### Effects of the GLCOM feature image alone with the rule-based plus ANN method

**Fig. 9:**
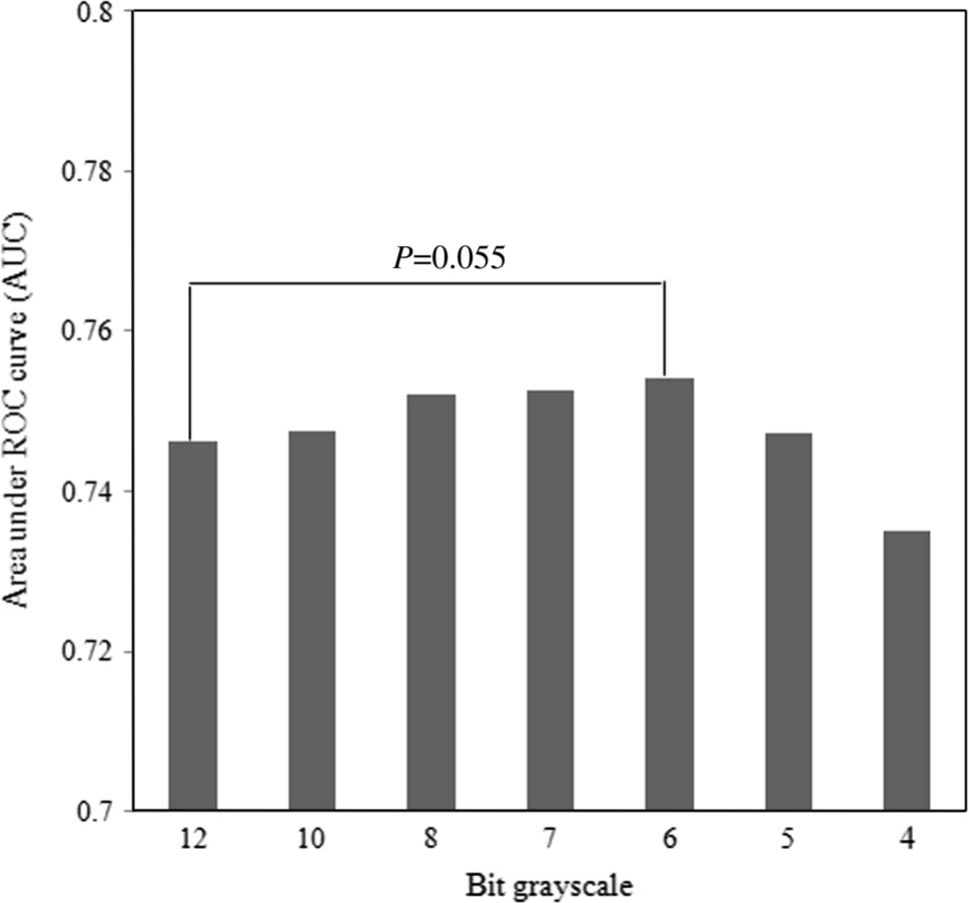
Effect of the gray-level scale

The effects of the gray-level scale on all small opacities are shown in Fig. 9. The AUC was 0.754 ± 0.083 for 6-bit depth, which was slightly larger than the value of 0.746 ± 0.097 for 12-bit depth (*P* = 0.055). There was no significant change in the classification performance. When the number of gray-level scales was reduced from 12-bit to 6-bit depth, there was no significant change in the classification performance. However, when the number of gray levels was reduced further to a 5-bit grayscale, the classification performance was degraded markedly. These results were similar to those of the previous study by Katsuragawa et al. [27]. They indicated that it was possible to represent lung texture patterns by a very small number of gray levels for computer analysis [27]. This somewhat unexpected result may be related to the fact that the basic pattern of the lung texture contains high PS values on low spatial frequency [27]. In addition, when a GLCOM feature image was obtained with high bit depth, because of the wide range of bit depths, the element of the GLCOM feature image was decreased and spread to various pixel values. On the other hand, when a GLCOM feature image was obtained with low bit depth, because the range of the bit grayscale was reduced, the element of the GLCOM feature image was increased and focused on some pixel value. Therefore, we selected a 6-bit depth in this study.

**Fig. 10:**
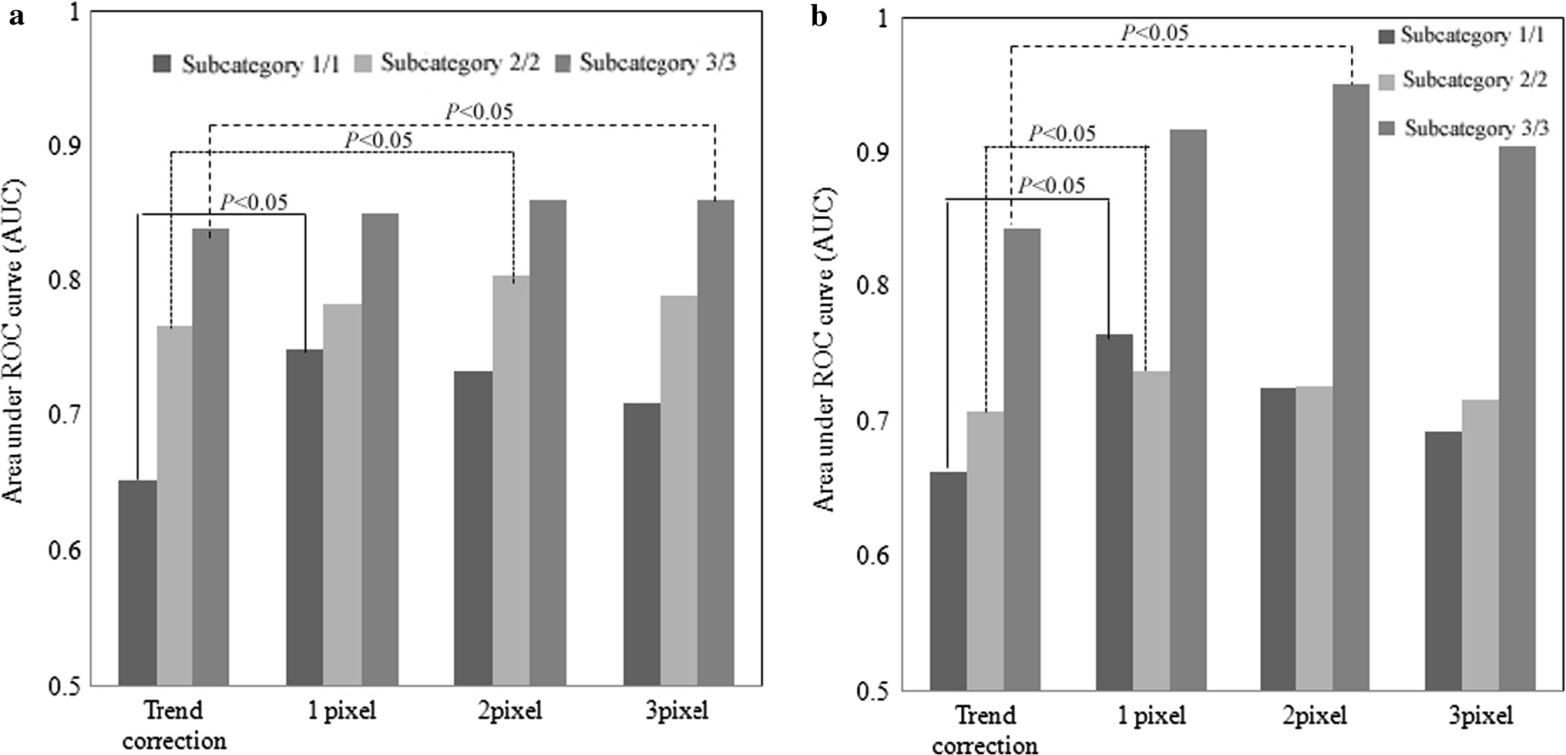
Effect of the GLCOM feature images on all small **a** rounded opacities and **b** irregular opacities

The effects of the GLCOM feature images on all small rounded opacities are shown in Fig. 10a. The AUC values in subcategories 3/3, 2/2, and 1/1 were 0.86 ± 0.04 with the GLCOM feature image (distance of 3 pixels), 0.80 ± 0.08 with the GLCOM feature image (distance of 2 pixels), and 0.75 ± 0.04 with the GLCOM feature image (distance of 1 pixel), respectively. The effects of the GLCOM feature images on all small irregular opacities are shown in Fig. 10b. The AUC values for subcategories 3/3, 2/2, and 1/1 were 0.95 ± 0.02 with the GLCOM feature image (distance of 2 pixels), 0.74 ± 0.05 with the GLCOM feature image (distance of 1 pixel), and 0.76 ± 0.05 with the GLCOM feature image (distance of 1 pixel), respectively. Relating the top-hat transform to the above, for subcategory 1/1 with a large distance between small opacities, we found that the distance of the GLCOM feature image was small. Therefore, for subcategory 1/1, when the values for two pixels between short distances were different, the element of the GLCOM feature image had a high value. This result suggested that the distances of 1 pixel on subcategory 1/1, 2 pixels on subcategory 2/2, and 3 pixels on subcategory 3/3 may correspond to the distance between small opacities.

### Classification performance by combined rule-based plus ANN method

Tables 3 and 4 show the AUC obtained with the classification method for small rounded opacities and irregular opacities, respectively. As shown in Tables 3 and 4, compared with the trend-correction image alone, the classification performance with the combined rule-based plus ANN method was improved in 12/15 (80.0 %) cases (*P* < 0.05). However, there was no statistical significance with other cases (3/15). Compared with the previous study, the classification performance was improved in all cases.

**Table 3:** Small rounded opacities with a size of *p*/*p* (a), *q*/*q* (b), and *r*/*r* (c) with the combined rule-based plus ANN method

	Trend correction alone	Combination analysis	*P* value
a			
Subcategory 1/1	0.61 ± 0.02	0.79 ± 0.02	<0.005
Subcategory 2/2	0.77 ± 0.03	0.85 ± 0.03	<0.005
Subcategory 3/3	0.8 ± 0.02	0.83 ± 0.02	<0.005
b			
Subcategory 1/1	0.64 ± 0.06	0.72 ± 0.05	<0.005
Subcategory 2/2	0.70 ± 0.04	0.76 ± 0.04	<0.005
Subcategory 3/3	0.84 ± 0.02	0.85 ± 0.03	0.18
c			
Subcategory^-^ 1/1	0.70 ± 0.04	0.76 ± 0.01	<0.005
Subcategory 2/2	0.83 ± 0.03	0.93 ± 0.03	<0.005
Subcategory 3/3	0.89 ± 0.02	0.90 **±** 0.01	0.056

*P* value: Student *t* test for paired data

**Table 4:** Small irregular opacities with a size of *s*/*s* (a) and *t*/*t* (b) with the combined rule-based plus ANN method

	Trend correction alone	Combination analysis	*P* value
a			
Subcategory 1/1	0.72 ± 0.04	0.78 ± 0.04	<0.005
Subcategory 2/2	0.72 ± 0.02	0.82 ± 0.04	<0.005
Subcategory 3/3	0.79 ± 0.04	0.91 ± 0.01	<0.005
b			
Subcategory 1/1	0.61 ± 0.04	0.72 ± 0.03	<0.005
Subcategory 2/2	0.69 ± 0.05	0.82 ± 0.04	<0.005
Subcategory 3/3	0.89 ± 0.04	0.93 ± 0.02	0.037

*P* value: Student *t* test for paired data

The combined rule-based plus ANN method with three enhancement methods showed the best classification performance for distinguishing between abnormal and normal ROIs. The three new enhancement methods decreased false-positive and false-negative ROIs. In addition, our results suggest that the rule-based plus ANN method with each of the three enhancement methods can complement each other. It should be noted that classification with the combined rule-based plus ANN method using the window function, top-hat transform, and GLCOM feature image provided the best performance.

On the cases with decreasing concentration of small opacities such as subcategory 1/1, the classification performance in the present study was slightly lower than in the previous studies [22, 28]. This is because the classification performance with the previous method was affected zone of the lung [22], or abnormal ROIs [28] included various subcategories, shapes, and sizes that it was easy for radiologists to classify as pneumoconiosis on chest radiographs. Therefore, for improved classification performance, typical texture patterns (each subcategory, shape, and size) were enhanced by texture features of the GLCOM, RLM. A gray-level ‘run’ was defined as set of consecutive pixels of the same gray level in a given direction. An element of the RLM measures the number of occurrence of a run with a specific length and specific gray level in a given direction [26]. Each ANN is trained independently for typical texture patterns (each subcategory, shape, and size). A multi-ANN [29] or first–third ANN [10] may show the highest classification performance. Our results were obtained for a relatively small number of cases (17 cases). Therefore, for evaluation of the clinical efficacy of this technique, a prospective study (ROC-type analysis) with large numbers of patients is required.

## Conclusions

We have developed a CAD system using three new enhancement methods for classification of pneumoconiosis on chest radiographs. The combined rule-based plus ANN method with window function, top-hat transform, and GLCOM feature image improved the classification performance in comparison with the rule-based plus ANN method. On the user interface for classification of pneumoconiosis on chest radiographs, in the future, square and circular markers will indicate normal and abnormal ROIs, respectively. The larger the circle, the greater the ANN output, which correspond to a greater abnormality. Thereby, our CAD system based on the new enhanced methods will be useful for assisting radiologists in the classification of the lowest subcategory (early pneumoconiosis) on chest radiographs.
